# An Unusual Case of Extramedullary Hematopoiesis in the Right Kidney

**DOI:** 10.7759/cureus.63924

**Published:** 2024-07-05

**Authors:** RB Revanth, Shivakumar S Swamy, Mahesh Ashok

**Affiliations:** 1 Onco-imaging, Healthcare Global Hospitals, Bangalore, IND; 2 Radiology, Healthcare Global Hospitals, Bangalore, IND

**Keywords:** renal tumour, polycythemia vera, renal mass, emh, extramedullary hematopoiesis

## Abstract

Extramedullary hematopoiesis (EMH) is the formation of blood cells outside the bone marrow, typically occurring in response to chronic anemia or bone marrow dysfunction. While EMH is most commonly observed in the liver, spleen, and lymph nodes, its occurrence in the kidney is exceedingly rare. In this case report, we are presenting a case of a 49-year-old male diagnosed with polycythemia vera who had an incidental right renal mass, which was histo-pathologically proven as extramedullary hematopoiesis in the right kidney mimicking lymphoma. This case underscores the importance of considering EMH in the differential diagnosis of renal masses, especially in patients with a history of myeloproliferative disorders. Early recognition and appropriate management are crucial to avoid unnecessary interventions and manage the underlying hematological condition effectively. Accurate diagnosis through histopathological examination is crucial to avoid unnecessary surgical interventions.

## Introduction

The generation of blood cells outside of the bone marrow, known as extramedullary hematopoiesis (EMH), occurs when the body is not producing enough blood cells. Leukemia, sickle cell disease, macrocytic anemia of hepatic origin, myelofibrosis, diffuse osseous metastatic disease, and thalassemia are the most frequent causes of EMH [1]. The liver, spleen, and lymph nodes are among the visceral organs that are typically affected by EMH. The pleura, lungs, gastrointestinal system, breast, skin, brain, kidneys, epidural space, and adrenal glands are less frequently affected [2]. EMH most commonly presents as paravertebral fat-containing masses, and it usually does not present a diagnostic dilemma. It is usually characterized by heterogeneous hypovascular soft-tissue masses that are frequently peppered with patches of attenuated fat on CT [3].

Renal involvement is extremely unusual and poses a diagnostic challenge because of its nonspecific presentation and potential for mimicking renal neoplasms. In this case report, we describe an unusual presentation of EMH in the right kidney, a location rarely documented in medical literature.

## Case presentation

A 49-year-old middle-aged man arrived at the hospital with complaints of on-and-off abdominal pain in the right lumbar region. Liver function tests and complete blood counts were done elsewhere in 2018 and showed elevated alkaline phosphatase and polycythemia. He was referred to healthcare global hospitals for imaging and further evaluation. General physical examination results were as follows: pulse, 80 bpm; blood pressure, 130/90 mm/hg; SPO_2_, normal. Moreover, no icterus, pallor, cyanosis, clubbing, lymphadenopathy, or edema was noted. On palpation of the abdomen, there was gross splenomegaly.

The patient was subjected to a blood test with the results as shown in Table 1.

**Table 1:** Lab reports.

Parameters	Observed value	Reference range
White Blood Cells (WBCs)	9,000/µL	4,000-11,000/µL
Red Blood Cells (RBCs)	4.2 million/µL	4.7-6.1 million/µL
Hemoglobin (Hgb)	9.3 g/dL	13.8-17.2 g/dL
Hematocrit (Hct)	33.1%	41-50%
Platelets	150,000/µL	150,000-450,000/µL
Total Bilirubin	0.8	0.1-1.2 mg/dL
Direct Bilirubin	0.2 mg/dL	0.0-0.3 mg/dL
Indirect Bilirubin	0.6 mg/dL	0.2-0.8 mg/dL
Alanine Aminotransferase (ALT)	25 U/L	7-56 U/L
Aspartate Aminotransferase (AST)	22 U/L	8-48 U/L
Alkaline Phosphatase (ALP)	141 U/L	44-147 U/L
Gamma-Glutamyl Transferase (GGT)	30 U/L	9-48 U/L
Total Protein	7.0 g/dL	6.3-8.3 g/dL
Albumin	4.5 g/dL	3.5-5.0 g/dL
Globulin	2.5 g/dL	2.0-3.5 g/dL
Albumin/Globulin Ratio	1.8	1.1-2.5
Serum Creatinine	0.9 mg/dL	0.6-1.2 mg/dL
Blood Urea Nitrogen (BUN)	15 mg/dL	7-20 mg/dL

On a peripheral smear, the red blood cells showed anisopoikilocytosis with normocytic normochromic, macrocytic, and many teardrop-shaped cells and normal morphology of WBCs and platelets. The impression was given as macrocytic anemia with many teardrop-shaped cells and a suspicion for myelofibrosis was raised. The patient also underwent urinalysis, which showed occasional mature urothelial cells and a few neutrophils. No malignant or RBCs were seen.

**Figure 1:**
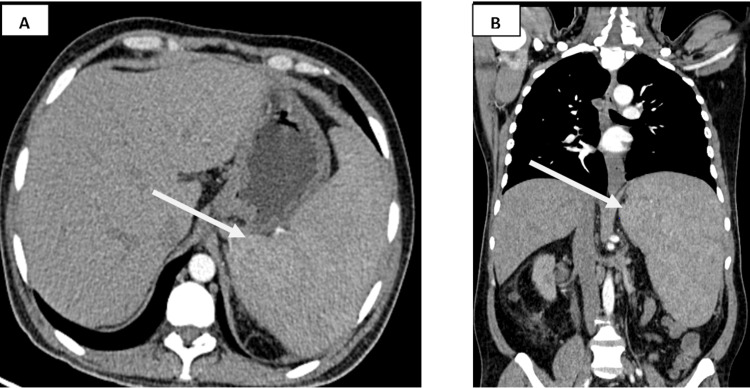
(A) Axial and (B) coronal reconstructed contrast-enhanced CT image in the arterial phase showing gross splenomegaly.

**Figure 2:**
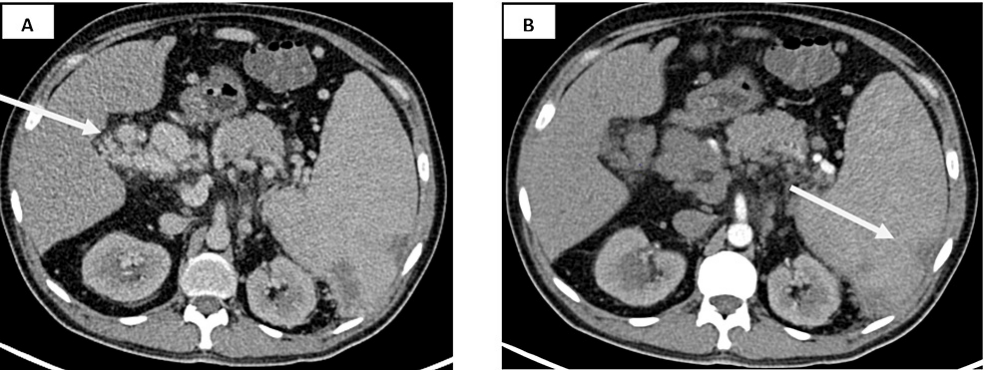
(A) Axial contrast-enhanced CT image showing numerous vascular structures adjacent to the portal vein, which enhance during the portal venous phase, suggestive of portal cavernoma. (B) Few nonenhancing hypodense-infarcted areas in the spleen.

**Figure 3:**
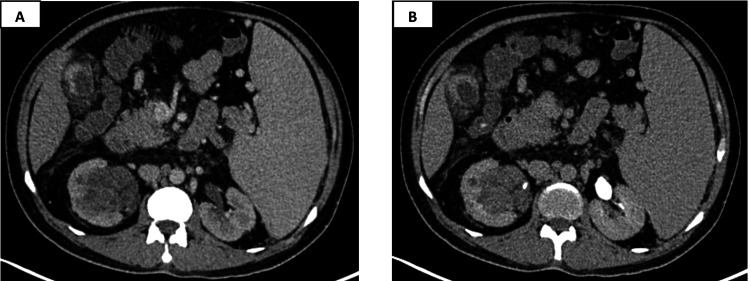
Contrast-enhanced CT images in venous (A) and delayed phases (B) showing a well-defined, solid, mildly enhancing lesion measuring 5.8 × 5.7 × 4.9 cm occupying the right renal pelvis causing a splaying of the collecting system.

The patient was then sent for contrast-enhanced CT (CECT) for further evaluation. On CT images, it was revealed that the patient had gross splenomegaly, portal cavernoma, and a mildly enhancing mass lesion in the right renal pelvis, suspicious for a right renal lymphoma. The CECT findings are shown in Figures 1-3.

**Figure 4:**
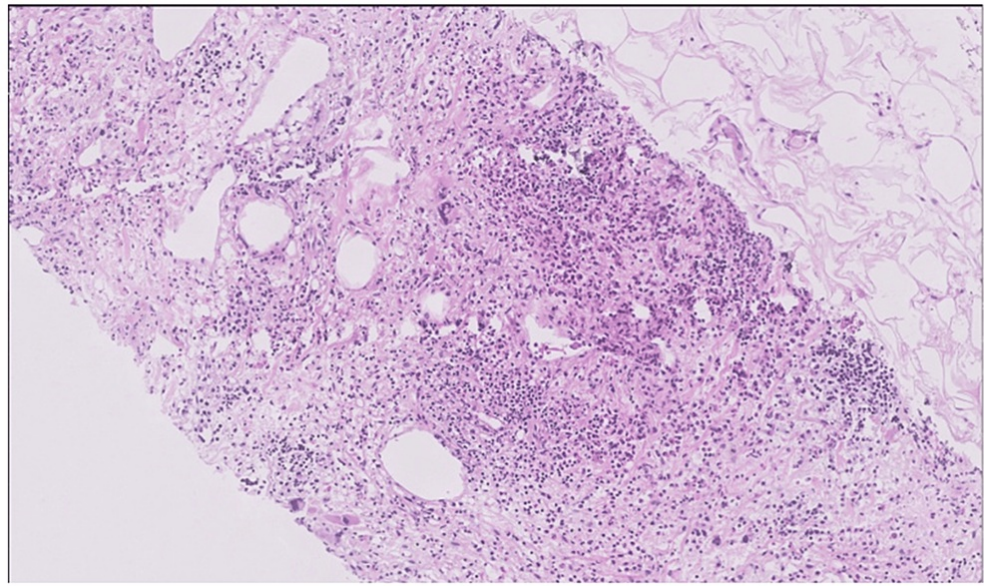
Histopathological image (10x) revealed cores of tissue showing extramedullary trilineage hematopoietic elements with full maturation (myeloid, erythroid, and megakaryocytic) admixed with mature adipose tissue and no atypical cells, consistent with EMH.

After the CECT study, the patient underwent ultrasound-guided biopsy of the right renal mass, which revealed cores of tissue showing extramedullary trilineage hematopoietic elements with full maturation (myeloid, erythroid, and megakaryocytic) admixed with mature adipose tissue and no atypical cells, consistent with EMH. Figure 4 shows the histopathological image. 

The patient then underwent a bone marrow biopsy, which revealed hypercellular marrow with trilineage proliferation with significant fibrosis, and was diagnosed with polycythemia vera, which had progressed to myelofibrosis.

A tailored approach was then used to treat the patient with a Janus kinase (JAK) inhibitor named ruxolitinib and supportive treatments, including erythropoiesis-stimulating agents (ESAs), namely, Epoetin alfa (Procrit, Epogen) and androgens, which help manage anemia. The patient is currently on regular monthly monitoring and is symptomatically better.

Although bone marrow transplantation offers a potential cure, it is reserved for select patients because of its associated risks and patient preference.

## Discussion

The kidney is an uncommon location for the incidence of EMH. There have been very few reported cases of renal EMH in the world. EMH in the kidneys grows slowly and is generally nondestructive, such as a benign tumor, and it tends to be encased in fibrous tissue. Renal involvement may be intrapelvic, perirenal, or parenchymal [4]. Generally, EMH shows variable enhancement patterns, but in our case, there was mild progressive enhancement with a mean Hounsfield unit (HU) value of 20 in a non-contrast study, 40 in the arterial phase, and around 60 in the delayed phase.

There can also be bilateral perirenal EMH which can mimic renal lymphoma. According to a study by Alexander et al. that examined kidney biopsy specimens from 1994 to 2015 and identified 14 patients with renal EMH, renal EMH should be considered when making a differential diagnosis of interstitial infiltrates, especially when glomerulopathy and hematologic malignancy are present [5]. Mubeen et al. reported a similar case in another case report, suggesting that the diagnosis of EMH should always be kept in mind before undergoing any drastic procedures and that CT-guided biopsy should be the first-line investigation [6]. In their case report, Koechale et al. came to the conclusion that patients with myelofibrosis are comparatively more likely to exhibit pelvicalyceal mass-like EMH on imaging. To definitively determine whether a correlation exists, more cases must be recorded and assessed. In our case, the lesion was found in the right renal pelvis, which was fairly similar to the cases reported [7].

Treatment options include numerous blood transfusions to reduce EMH, and JAK inhibitors such as ruxolitinib and fedratinib, which are commonly used for their efficacy in symptom control and spleen size reduction. Supportive treatments, including erythropoietin-stimulating agents and androgens, help manage anemia, while hydroxyurea and interferons can also be used to control blood counts and disease progression [8].

## Conclusions

One should consider EMH as a differential diagnosis when dealing with renal masses, especially in the background of a hematological disease. The location of the mass in the renal pelvis may be specific for myelofibrosis. Although there is no specific pathognomonic finding to diagnose EMH in the kidney, imaging aids in locating and biopsying the lesion, whereas histopathology is essential for accurately diagnosing it and differentiating it from other renal masses, thereby helping prevent needless nephrectomies.
